# Suspected anaphylaxis during anesthesia induction without identified allergens: a case report

**DOI:** 10.1186/s40981-023-00684-y

**Published:** 2023-12-18

**Authors:** Sayaka Hirai, Mitsuru Ida, Ai Arima, Masahiko Kawaguchi

**Affiliations:** 1 Department of Anaesthesiology, Nara Medical University, Kashihara, Nara Japan; 2 Department of Dermatology, Nara Medical University, Kashihara, Nara Japan

To the Editor,

The Japanese Society of Anesthesiologists offers practical guidelines for dealing with perioperative anaphylaxis, emphasizing the importance of anesthesiologists’ involvement in identifying the causative agent to prevent recurrence [1]. However, identifying the causative agents is not always feasible. Herein, we report, with written informed consent, a case where anaphylaxis was suspected during anesthesia induction, yet no allergens were identified.

A 59-year-old man, 165.5 cm in height and weighing 65.1 kg, presented with congestive heart failure, chronic kidney disease, diabetes, hypertension, and hyperlipidemia, requiring coronary artery bypass grafting for triple-vessel coronary artery disease. The patient had not undergone any surgery previously and had not taken any angiotensin receptor blockers and angiotensin-converting enzyme inhibitors. In the operating room, standard vital signs were closely monitored, and non-invasive blood pressure (NIBP) was recorded at 160/120 mmHg. Anesthesia was induced using remifentanil (rate, 20 mL/h) and remimazolam (12 mg/kg/h). Upon confirming the loss of consciousness, the dosages of remifentanil and remimazolam were reduced to 5 mL/h and 1.0 mg/kg/h, respectively, four minutes after administering rocuronium (60 mg). This was followed by tracheal intubation and arterial catheter insertion. His blood pressure (BP) was 89/67 mm Hg (NIBP) and 47/25 mm Hg (arterial line) immediately before and after tracheal intubation, respectively. Despite fluid resuscitation of 500 mL and multiple boluses of ephedrine (16 mg), phenylephrine (0.3 mg), and norepinephrine (10 µg), he experienced cardiac arrest. During chest compressions, an intravenous bolus of epinephrine (0.1 mg) was administered, resulting in cardiopulmonary resuscitation with an arterial BP of 46/29 mmHg. However, due to persistent severe hypotension, continuous infusions of norepinephrine at 0.1 mcg/kg/min and dobutamine at 5 mcg/kg/min were initiated following additional boluses of epinephrine (0.3 mg). Figure 1 displays the patient’s vital signs during anesthesia. Edema with erythema of the extremities and trunk was observed throughout this sequence, and transesophageal echocardiography revealed no evidence of cardiogenic shock. Consequently, anaphylaxis was suspected, and the patient was transferred to the intensive care unit without proceeding with surgery. Blood samples taken before he left the operating room indicated an elevated serum tryptase level of 17.1 μg/L, exceeding the normal range of 1.2–5.7 μg/L. More than seven weeks after the onset, both basophil activation and skin prick tests using remimazolam and rocuronium yielded negative results. The patient declined surgery and was subsequently followed-up after percutaneous coronary intervention at coronary segments 6, 7, 11, and 14.

**Fig. 1:**
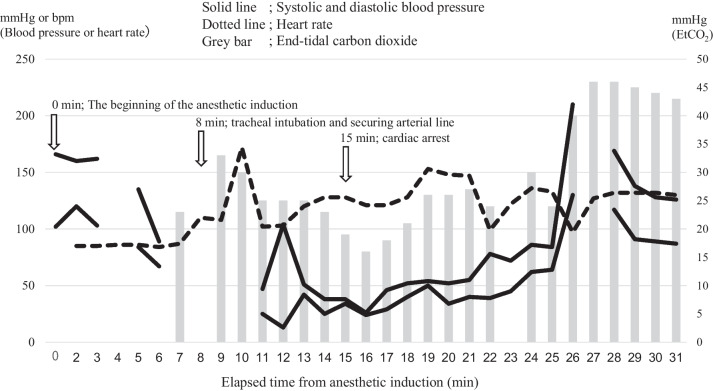
The patient’s vital signs during the anesthetic. 0 min, the beginning of anesthetic induction; 3 min, remimazolam and remifentanil were initiated; 4 min, rocuronium was administrated; 8 min, tracheal intubation and securing arterial line; 9 min, phenylephrine 0.1 mg; 10 min, ephedrine 8 mg; 11 min, remimazolam and remifentanil were discontinued; 13 min, phenylephrine 0.2 mg; 14 min, ephedrine 8 mg; 17 min, adrenaline 0.1 mg; 23 min, norepinephrine 0.1 mcg/kg/min, dobutamine 5 mcg/kg/min, and adrenaline 0.3 mg; 28 min, adrenaline 0.3 mg; 30 min, adrenaline 0.3 mg and hydrocortisone 100 mg; 31 min, famotidine 20 mg and hydroxyzine 25 mg. There were no data regarding blood pressure from 6 to 11 minutes after anesthesia induction, as non-invasive blood pressure monitoring was discontinued 6 minutes after anesthesia induction because we expected that it would be replaced by arterial blood pressure monitoring

Identifying the causative agents is crucial, although it is important to acknowledge the inherent risks [1]. Given the patient's age and the elevated risk of myocardial ischemia, tests were conducted in anticipation of future anesthesia. However, dermatologists opted against an intradermal test due to hypotension preceding skin symptoms during anesthetic induction. Skin tests are considered the gold standard for immunoglobulin E (IgE)-mediated anaphylaxis detection [2]. Considering the patient’s negative results, it is presumed that the anaphylactic reaction was non-IgE-mediated [3]. In this case, serum tryptase levels were measured only once, and the allergens were not identified. This may indicate that the hypotension during anesthetic induction was not due to an allergic reaction; however, the edema with erythema of the extremities and trunk cannot be explained by hypotension caused by excessive anesthetics. Anaphylaxis was suspected during anesthetic induction, and attempts were made to identify the causative agent. Unfortunately, the examination concluded without identifying the suspected drug. Prevention of recurrent anaphylaxis is possible by avoiding the suspect drug. However, continued contraindication of a key agent in general anesthesia, such as rocuronium, is a significant disadvantage for both the anesthesiologist and the patient. Therefore, anesthesiologists should make every effort to identify the causative agent of anaphylaxis.

## Data Availability

Not applicable.

## Abbreviations

BP: Blood pressure
IgE: Immunoglobulin E
NIBP: Non-invasive blood pressure
